# Distinguishing Glioblastoma Subtypes by Methylation Signatures

**DOI:** 10.3389/fgene.2020.604336

**Published:** 2020-11-24

**Authors:** Yu-Hang Zhang, Zhandong Li, Tao Zeng, Xiaoyong Pan, Lei Chen, Dejing Liu, Hao Li, Tao Huang, Yu-Dong Cai

**Affiliations:** ^1^School of Life Sciences, Shanghai University, Shanghai, China; ^2^Channing Division of Network Medicine, Brigham and Women’s Hospital, Harvard Medical School, Boston, MA, United States; ^3^College of Food Engineering, Jilin Engineering Normal University, Changchun, China; ^4^Shanghai Research Center for Brain Science and Brain-Inspired Intelligence, Shanghai, China; ^5^Institute of Image Processing and Pattern Recognition, Shanghai Jiao Tong University, and Key Laboratory of System Control and Information Processing, Ministry of Education of China, Shanghai, China; ^6^College of Information Engineering, Shanghai Maritime University, Shanghai, China; ^7^Shanghai Institute of Nutrition and Health, Shanghai Institutes for Biological Sciences, Chinese Academy of Sciences, Shanghai, China

**Keywords:** glioblastoma, methylation, signature, subtype, classification

## Abstract

Glioblastoma, also called glioblastoma multiform (GBM), is the most aggressive cancer that initiates within the brain. GBM is produced in the central nervous system. Cancer cells in GBM are similar to stem cells. Several different schemes for GBM stratification exist. These schemes are based on intertumoral molecular heterogeneity, preoperative images, and integrated tumor characteristics. Although the formation of glioblastoma is remarkably related to gene methylation, GBM has been poorly classified by epigenetics. To classify glioblastoma subtypes on the basis of different degrees of genes’ methylation, we adopted several powerful machine learning algorithms to identify numerous methylation features (sites) associated with the classification of GBM. The features were first analyzed by an excellent feature selection method, Monte Carlo feature selection (MCFS), resulting in a feature list. Then, such list was fed into the incremental feature selection (IFS), incorporating one classification algorithm, to extract essential sites. These sites can be annotated onto coding genes, such as *CXCR4, TBX18, SP5*, and *TMEM22*, and enriched in relevant biological functions related to GBM classification (e.g., subtype-specific functions). Representative functions, such as nervous system development, intrinsic plasma membrane component, calcium ion binding, systemic lupus erythematosus, and alcoholism, are potential pathogenic functions that participate in the initiation and progression of glioblastoma and its subtypes. With these sites, an efficient model can be built to classify the subtypes of glioblastoma.

## Introduction

Glioblastoma, also called as glioblastoma multiform (GBM), is the most aggressive cancer that initiates within the brain. The cause of this disease is unclear. The risk factors of GBM include genetic factors and environmental factors, such as smoking and exposure to pesticides. Similar to other brain cancers, GBM can cause epilepsy, nausea, vomiting, headaches, and mild hemiplegia. The typical symptoms of glioblastoma are deteriorating memory and personality or decline in neurological function. Most symptoms are caused by the destruction of the temporal lobes and the frontal lobes. Different subspecies of glioblastomas are produced in the central nervous system, and cancer cells in GBM are similar to stem cells.

Several different schemes for glioblastoma stratification exist. One is based on intertumoral molecular heterogeneity in GBM. This scheme identities the subtypes of procedural and mesenchymal glioblastoma on the basis of the biomarker genes *VEGF-A*, *VEGF-B*, *ANG1*, and *ANG2* (Sharma et al., 2017). The second technique involves the use of preoperative images as predictive markers of GBM subtypes; in this approach, the distinctive imaging phenotypes and imaging patterns of glioblastoma subtypes are detected by employing machine-learning techniques (Macyszyn et al., 2016). The third technique is based on integrated tumor subtypes, which have been discovered through an integrative subtype analysis of the GBM dataset from the cancer genome atlas (TCGA) (Shen et al., 2012).

The promoter region is a functional part of the genome that is regulated by methylation and contributes to the regulation of gene expression during the pathogenesis of glioblastoma. Such genomic modification affects the expression of a group of important proteins, including *MGMT*, *GATA6*, and *CASP8*; the dysmethylation of these genes is remarkable in glioblastoma (Skiriute et al., 2012). For example, through whole-genome wide methylation screening, a study found that 5 m-dC level is the best discriminant among methylation classes, and the upregulation of *LINE1* methylation is an independent prognostic factor in GBM diseases (Lai et al., 2014). Although the formation of glioblastoma is related to gene methylation, glioblastoma has been poorly classified on the basis of epigenetics.

Preliminary attempts on clustering GBMs using epigenetic biomarkers have already started. According to a systematic analysis on the DNA methylation-based classification of central nervous system tumors (Guardiola Bagán et al., 2017; Capper et al., 2018), central nerve system (CNS) tumors can be further classified into multiple subgroups based on the whole-genome wide methylation status. As one important part of the CNS tumors, GBM can be further classified into eight classes, which is DMG K27, GBM G34, GBM MES, GBM RTK I, GBM RTK II, GBM RTK III, GBM MID, and GBM MYCN. Researchers tried to use unsupervised clustering of reference samples using t-SNE dimensionality reduction. According to the original publications, group DMG K27 can be easily distinguished from other seven groups based on the results of t-SNE based separation. However, the differences between the other seven subgroups cannot be clarified clearly and the specific methylation locus that contribute to the separation have not been identified. Therefore, in this study, we used methylation datasets downloaded from Gene Expression Omnibus (GEO) database to identify specific methylation locus/biomarkers that contribute to the classification and annotation of different GBM subgroups (Capper et al., 2018).

We aimed to identify essential methylation sites (features) in this study, on which the subtypes of glioblastoma can be efficiently classified. To this end, we employed two datasets collected in GEO. One dataset was termed as the training dataset, whereas the other was treated as the independent test dataset. A powerful feature selection method, Monte Carlo feature selection (MCFS) (Dramiński et al., 2007), was applied on the training dataset. A feature list, indicating the importance of features, was produced. After that, incremental feature selection (IFS) (Liu and Setiono, 1998) was executed on this list, which incorporated one classification algorithm, to extract essential methylation sites. As a result, we found 4100 methylation sites (features) associated with the classification of GBM. These sites can be annotated onto coding genes, such as *CXCR4, TBX18, SP5*, and *TMEM22*. Through the further functional enrichment analysis of these dysmethylated genes using GO and KEGG databases, we identified several biological functions related to GBM classification (e.g., subtype-specific functions). Also, with these methylation sites, an efficient model with support vector machine (SVM) (Cortes and Vapnik, 1995) as the prediction engine can be built to classify subtypes of glioblastoma. In summary, on the basis of the powerful computational approaches, we identified various novel potential pathogenic genes at the epigenetics level and revealed several potential pathogenic functions that participate in the initiation and progression of glioblastoma and its subtypes with wide support from recent reports.

## Materials and Methods

### Dataset

Two sets of methylation profiles of patients with GBM were downloaded from GEO with the accession numbers GSE90496 and GSE109379 (Capper et al., 2018). The first dataset included 347 GBM cases and the second dataset contained 324 GBM cases. These two datasets were used as the training dataset and independent test dataset, respectively. All GBM cases are classified into seven categories. The distribution of GBM cases on seven categories is listed in Table 1. The methylation levels of 42,383 probes were used to represent each patient. The goal was to identify discriminative methylation features (e.g., dysmethylated sites or genes) corresponding to different GBM subtypes.

**TABLE 1:** Breakdown of the GBM samples in the training and independent datasets.

Category	Training dataset	Independent dataset
G34	41	13
MES	56	104
MID	14	19
MYCN	16	17
RTK	64	44
RTK II	143	118
RTK III	13	9

### Feature Selection

In this study, we first used MCFS (Dramiński et al., 2007) to identify the general interpretable information of features (methylation sites) in tumor samples from the central nervous system. Then, we applied IFS (Liu and Setiono, 1998) to improve classification performance by obtaining a group of optimal features with the strong recognition ability of central nervous system tumors.

#### MCFS

Monte Carlo feature selection is a classical and powerful feature selection method wherein decision trees are used to find distinguishable features for classification (Dramiński et al., 2007). It is quite suitable to analyze datasets with features much more than samples. The datasets described in section “Dataset” are in such type. Thus, we adopted MCFS to analyze the training dataset, aiming to extract essential features. Furthermore, such feature selection method can deeply investigate complicated relationship between features or class labels, extracting essential features in deep levels.

The MCFS method evaluates the importance of features by constructing lots of decision trees. Given a dataset with *M* features, randomly construct *s* feature subsets consisting of *m* features, where *m* is much smaller than *M*. For each feature dataset, *t* bootstrap sample sets are constructed from the original dataset, in which samples are represented by features in such feature subset. Accordingly, *t* decision trees are built. After all feature subsets are processed by the above procedures, *s*⋅*t* decision trees are constructed. Based on these trees, a feature *g* is assigned a relative importance (RI) value, which can be calculated by

(1) $$R\,I_{g}=\sum_{\tau =1}^{s\,t}{\left(w\,A\,c\,c\right)}^{u}\,\sum_{n_{g}\,\left(\tau \right)}I\,G\,\left(n_{g}\,\left(\tau \right)\right)\,{\left(\frac{\mathrm{no}.\mathrm{in}\,n_{g}\,\left(\tau \right)}{\mathrm{no}.\mathrm{in}\,\tau }\right)}^{v},$$

where *IG*(*n*_*g*_(τ)) stands for the gain information of node *n*_*g*_(τ), (no. in *n*_*g*_(τ)) represents the number of samples in node *n*_*g*_(τ), (no. in τ) denotes the number of samples in tree τ, *wAcc* indicates the weighted accuracy of the tree. *u* and *v* are the regular factors, which were suggested to set to one (Dramiński et al., 2007). All investigated features are ranked in a list with the decreasing order of their RI values. Clearly, features with high ranks are more important than those with low ranks.

In present study, we used the MCFS program retrieved from http://www.ipipan.eu/staff/m.draminski/mcfs.html. Default parameters were adopted.

#### IFS

Incremental feature selection is a feature selection method used to distinguish between samples from different classes (e.g., normal and diseased) (Liu and Setiono, 1998). In this study, different classes of samples were discerned by a set of optimal features screened by IFS performed in a rank-descending feature list. We set candidate high-performance feature subsets as feature subsets with large interval sizes (e.g., 10 features) from the ranked feature list. Suppose *N* candidate feature subsets F = [*F*^1^,*F*^2^,…,*F^N^*] exist. The *i*-th feature subset includes 10 * *i* features yielding *F^i^* = [*f*_1_,*f*_2_,…,*f*_i*N_]. We construct and evaluate the classifier on each candidate feature subset. The candidate feature subset with the maximal prediction performance is the optimal feature subset, and the classifier constructed from these optimal features is the optimal classifier.

### Classification Algorithm

#### Support Vector Machine

The classifier acts as a classification model that maps data samples to a given category for data class prediction. We use support vector machine (SVM) (Cortes and Vapnik, 1995) based on statistical learning theory for supervised data classification. It has wide application for tackling different biological problems (Muthukrishnan et al., 2014; Chen et al., 2017, 2020; Liu et al., 2020; Sang et al., 2020; Zhou et al., 2020a,b). The basic principle is to use a given kernel function (e.g., Gaussian kernel) to transform data from a low-dimensional space to a high-dimensional space. The SVM model can separate the samples of each class/category by maximizing the data interval and also predicts (new) sample categories on the basis of the interval where this sample falls in. For two-class classification, the largest margin between the two categories of samples can be inferred by SVM, where large margins are associated with small generalization error. For multiclass classification, SVM uses the “One Versus the Rest” strategy. In this study, we solved the optimization problem of SVM by using the sequence minimization optimization (SMO) algorithm (Platt, 1998; Keerthi et al., 2001) implemented by the tool “SMO” in Weka software (Frank et al., 2004; Witten and Frank, 2005), which can be downloaded at https://www.cs.waikato.ac.nz/ml/weka/. For convenience, the default parameters were adopted, where the kernel was a polynomial function and the regularization parameter *C* was set to one.

#### Random Forest

A random forest (RF) (Breiman, 2001) is a metaclassifier that contains a large number of tree classifiers for establishing final joint classification, which determines the output categories/classes by summarizing votes from different decision trees (Breiman, 2001). The RF is a commonly used method in machine learning and is widely applied in computational biology (Pan et al., 2010; Zhao et al., 2018; Jia et al., 2020; Liang et al., 2020; Yuan et al., 2020). Notably, a slight difference exists between each decision tree and other decision trees in a RF. Thus, the predictions of all decision trees are averaged to obtain the final decision of RF. This approach can avoid over-fitting and improve the performance of the integrated model. However, it slightly increases the bias of the overall model and causes the loss of some interpretability. In this study, we used the tool “RandomForest” in Weka (Frank et al., 2004; Witten and Frank, 2005), which implemented the above RF. The number of decision trees was set to ten.

#### Rule Learning

In this study, we used the rule learner known as repeated incremental pruning to produce error reduction (RIPPER) to generate classification rules for classifying samples from different GBM subtypes (Cohen, 1995). RIPPER learns interpretable classification rules consisting of IF–ELSE rules. Briefly, RIPPER learns the rules of one class and then moves to learn the next class in a given order, e.g., it learns from the first minority class to the next until the dominant class. To quickly implement the RIPPER algorithm, we directly employed the tool “JRip” in Weka (Frank et al., 2004; Witten and Frank, 2005). Default parameters were used.

### Functional Enrichment Analysis

The selected optimal methylation probes (features) were mapped onto genes on the basis of the annotation files of GPL13534 downloaded from GEO. The enrichments of these genes on GO terms and KEGG pathways were evaluated with hypergeometric tests measured by phyper function in R^1^. The cutoff of the adjusted hypergeometric test *p*-values, i.e., FDR (false discovery rate), was set to 0.05. In other words, only the GO terms and KEGG pathways with FDR < 0.05 were considered to be statistically significant.

### Performance Measurement

We employed Matthew Correlation Coefficients (MCC) (Matthews, 1975; Gorodkin, 2004) to evaluate the performance metrics of different kinds of classifiers. The MCC accounts for true and false positives and true and false negatives, and this measurement has values ranging from −1 and +1. It is a common method for calculating the correlation between target and prediction classes. Applying 10-fold cross-validation (Kohavi, 1995), we used MCC to evaluate the performance of different training models for glioblastoma classification.

## Results

**FIGURE 1:**
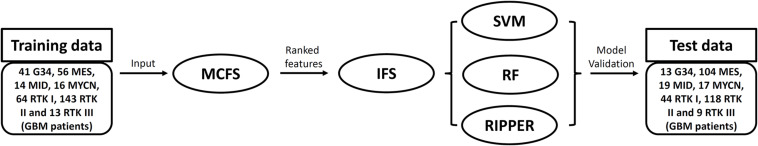
Flowchart of the analysis performed in this study. The training dataset is first analyzed by the Monte Carlo feature selection (MCFS) method. Features are ranked in a list, which is fed into the incremental feature selection (IFS) with one of three classification algorithms. The optimal classifiers based on different classification algorithms are built and further evaluated their performance on a test dataset.

In this study, we investigated the methylation profiles of GBM patients. The entire procedures are illustrated in Figure 1.

### Results of MCFS Method on the Training Dataset

We first used MCFS to analyze the training dataset. Each feature was evaluated by a RI value. Accordingly, all features were ranked in the decreasing order of their RI values. Obtained feature list is provided in Supplementary Table 1.

### IFS Results

**FIGURE 2:**
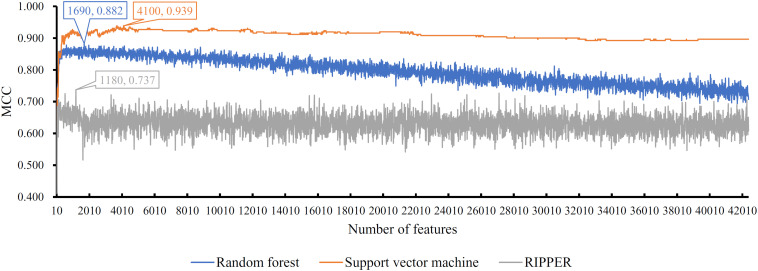
IFS curves with support vector machine, random forest, and RIPPER on the training set. The support vector machine can yield the highest MCC (0.939) when top 4100 features are used, while the highest MCCs of random forest and RIPPER are 0.882 and 0.737, respectively, when top 1690 and 1180, features respectively, are adopted.

**FIGURE 3:**
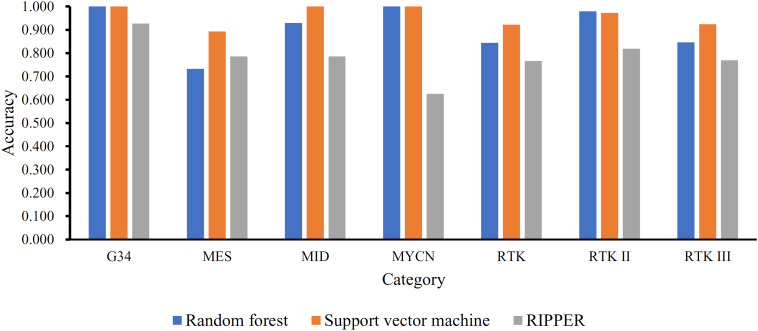
Performance of the optimal SVM, RF, and RIPPER classifiers on different categories in the training dataset. The optimal SVM and RF classifiers are much superior to the optimal RIPPER classifier, and the optimal SVM classifier is slightly superior to the optimal RF classifier.

Next, we generated a series of feature subsets from the MCFS feature list and then subjected them to IFS with SVM, RF, and RIPPER to obtain the best features for classifying different categories of GBM samples. The complete results of the three classifiers using different number of features are given in Supplementary Table 2. For an easy observation, an IFS curve was plotted with number of used features as X-axis and MCC as the Y-axis for each classification algorithm, as shown in Figure 2, in which the highest MCC of each classification is marked. It can be observed that the highest value of MCC generated by SVM was 0.939 when using the top-ranked 4100 features. Accordingly, we constructed the optimal SVM classifier with these 4100 features. For RF, when using the top-ranked 1690 features, the largest MCC value of 0.882 was achieved. These 1690 features were used to build the optimal RF classifier. When using the top-ranked 1180 features, the highest MCC value of 0.737 was obtained by RIPPER. The optimal RIPPER classifier was built based on these 1180 features. The overall accuracies of above-mentioned classifiers are listed in Table 2 and the accuracies on seven categories are shown in Figure 3. As shown by these results, the optimal classifier was SVM, which was superior to RIPPER and RF although it used additional features.

**TABLE 2:** 10-fold cross-validation performance of the optimal SVM, RF, and RIPPER classifiers on the training set.

Classification algorithm	Number of features	Overall accuracy	MCC
SVM	4100	0.954	0.939
RF	1690	0.911	0.882
RIPPER	1180	0.804	0.737

### Performance of Optimal Classifiers on the Test Dataset

**FIGURE 4:**
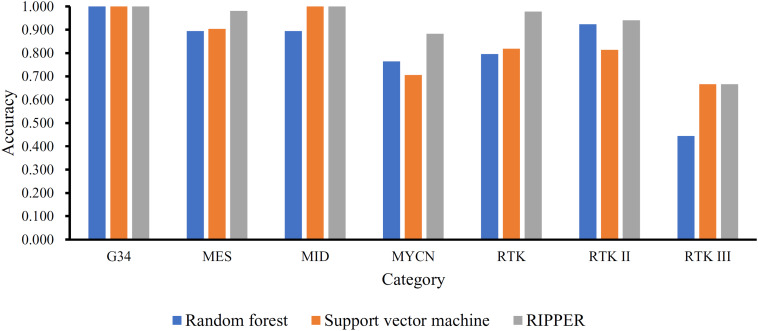
Performance of the optimal SVM, RF, and RIPPER classifiers on different categories in the independent test dataset. The optimal RIPPER classifier gives the best generalizability on the independent test dataset, followed by the optimal RF and SVM classifier.

To show the generalizability of our pipeline, we also evaluated above-constructed classifiers on a completely independent test dataset. The MCCs generated by the optimal SVM, RF, and RIPPER classifiers were 0.798, 0.832, and 0.937. These results are summarized in Table 3, in which the corresponding overall accuracies are also listed. The detailed performance on each category is shown in Figure 4. The results indicated that the RIPPER classifier had better generalizability than other two algorithms, and SVM shown the worst generalizability performance in this study.

**TABLE 3:** Performance of the optimal SVM, RF, and RIPPER classifiers on the independent test dataset.

Classification algorithm	Overall accuracy	MCC
SVM	0.852	0.798
RF	0.877	0.832
RIPPER	0.954	0.937

### Results of Enrichment Analysis

On the training dataset, the optimal SVM classifier gave the best performance, which adopted 4100 top-ranked features (methylation sites). These sites were mapped onto genes based on the annotation file of Illumina HumanMethylation450 BeadChip from GEO with platform number of GPL13534^2^, resulting in 1813 coding genes, which are provided in Supplementary Table 3. For consistency, these genes were called optimal genes in the following text.

The enrichment analysis was done on the above 1813 genes. The results are listed in Supplementary Table 4. Several GO terms and KEGG pathways with FDR < 0.05 were obtained. In detail, we obtained 167 biological process (BP) GO terms, 28 cellular component (CC) GO terms, 26 molecular function (MF) GO terms and four KEGG pathways. Some of them would be analyzed in section “Biological Functions Relevant to GBM Based on Optimal Genes” and “Biological Pathways Relevant to GBM Based on Optimal Genes.”

## Discussion

### Optimal Genes Relevant to GBM

As mentioned in section “Results of Enrichment Analysis,” 1813 optimal genes were obtained. We selected some of them for analysis in this section. These genes are targeted by probes with high RI values.

The first gene is ***CXCR4*** (targeted by probe **cg02902079** and **cg10824187**), which is a lymphocyte activity regulation molecule and acts as an alpha-chemokine receptor specific for stromal-derived-factor-1. Chemokines play important autocrine and paracrine roles during tumor initiation and progression. Generally, the *in vivo* secretion of chemokines regulates the biological effects of various components in the microenvironment of *CXCR4* (Würth et al., 2014). In cancer stem cells, *CXCR4* is upregulated and plays an irreplaceable role in perivascular invasion, a specific tumor behavior in GBM (Yadav et al., 2016). In addition, *CXCR4* is an effective target for improving tumor sensitivity in GBM in conjunction with radiation therapy (Yadav et al., 2016). Moreover, *CXCR4* is suppressed by *PATZ1*, which is enriched in the proneural subtype and colocalizes with stemness markers of GBMs (Guadagno et al., 2017).

The next identified probe turns out to be **cg26558485**, targeting the 5′UTR of CYP4X1. As a member of the cytochrome P450 superfamily of enzyme, such gene has been generally reported to participate in neurovascular function in the brain (Bylund et al., 2002). As for its correlations with GBM, recently, two successive related publications (Wang et al., 2018, 2019) confirmed that CYP4X1 contributes to the inhibition of glioma angiogenesis. Glioma vasculature is quite significant for the initiation and progression of such disease (Hardee and Zagzag, 2012). The methylation of related functional regions of such gene definitely affect its biological functions, which further plays an irreplaceable role for GBM pathogenesis. Therefore, such target gene can be an effective GBM associated gene.

Apart from probe **cg26558485**, another probe named as **cg07028914** targets a transcription factor named as ***TBX18***. According to recent publications, an independent study in 2015 confirmed that microRNA miR-205 prevent the invasion of glioma by targeting *TBX18* (Zheng et al., 2015), reflecting the potential regulatory role of *TBX18* during glioma pathogenesis. Most of microRNAs’ biological effects on glioma pathogenesis relied on the regulation on gene expression, which is similar with methylation mediated biological processes. Therefore, the methylation status of such gene may also play a potential regulatory role for the invasion of glioma.

The next gene, ***SP5*** is targeted by multiple probes including **cg26766005** and **cg14768335**. According to recent publications, *SP5* has been shown to be therapeutic target and a prognostic biomarker for multiple cancer subtypes, including glioma (Safe and Abdelrahim, 2005; Safe et al., 2014). Considering that methylation can regulate the expression level and biological effects of a target gene, the methylation status of the regulatory region of such gene may also probably affect the pathogenesis of glioma and have different pathological effects in different glioma subgroups indirectly.

As for ***TMEM22***, also known as *SLC35G2*, which is targeted by the optimal features **cg25836094, cg13383019**, and **cg22304507**, it has been generally reported to participate in cell proliferation and tumorigenesis with few publications (Dobashi et al., 2009). Although such gene has not been directly reported to be functionally correlated with glioma, it has been widely reported to be associated with renal cell carcinoma and its homolog which shared similar biological functions, *TMEM97* has been directly confirmed to be correlated with glioma at transcriptomics level. Considering that methylation at gene body is correlated with gene transcription, it is reasonable for us to regard *TMEM22* associated probes as potential glioma associated probes.

The next identified probe turns out to be **cg11823511**, targeting gene ***BARHL2***. According to two independent studies reported by researchers from University of Birmingham (Dunwell et al., 2010) and Memorial Sloan-Kettering Cancer Center (Shen et al., 2012), respectively, the methylation of *BARHL2* is not only related to hematological and epithelial cancers, but nerve system malignancies including glioma and may play a specific role for the integrative subgrouping of glioma (Shen et al., 2012).

***RASGRF2*** targeted by probe **cg06829830** has also been predicted to be contribute to the pathogenesis of glioma at methylation level. According to recent publications, in 2019, a systematic review (Wu et al., 2014) on the cancer methylation biomarkers confirmed that such gene is a specific biomarker for aggressive gliomas at methylation level using liquid biopsy.

Apart from such gene, the next identified biomarker is ***TLX3***, targeted by probe **cg26844246**. The methylation alteration of such gene has been identified in multiple tumor subtypes, like thyroid cancer (Kikuchi et al., 2013), bladder cancer and lung adenocarcinoma (Pradhan et al., 2013). In a systematic study on the whole-genome wide glioma methylation status, *TLX3* has been shown with specific methylation status in level II and III gliomas (Suzuki et al., 2015).

As for gene ***ANKRD34A*** (correlated with probes **cg10178263, cg18280463**, and **cg13947666**), according to related methylation studies (Giri and Aittokallio, 2018; Ding et al., 2020), such gene has shown to have methylation changes during the initiation and progression of multiple tumor subtypes, including lung, colon, bladder, lymphoma, breast and ovarian cancer. Therefore, it is reasonable for us to connect the methylation status of *ANKRD34A* with glioma. Apart from that, a recent publication (Ding et al., 2020) in 2020 also indicated that the transcript of such gene, which is regulated by methylation status, may participate in the RNA regulatory network in low grade glioma. Therefore, the methylation of such gene may be correlated with glioma and performed differentially in different subgroups.

The last target of the optimal probes is ***MARCH11*** (targeted by probe **cg09017434)**, regulating the intracellular transport of lysines. As for its correlations with GBM, according to recent publications, such gene has shown to be correlated with the carcinogenic transformation of cells with different expression levels (Yang et al., 2020). Considering the correlations between gene region methylation and gene expression, it is reasonable for us to speculate that the methylation status of such gene may be correlated with potential malignant alterations, supporting its correlations with GBM.

### Biological Functions Relevant to GBM Based on Optimal Genes

Here, to summarize the specific biological functions that may contribute to revealing the differences between different GBM subgroups at methylation level, we performed GO enrichment analyses and pathway analyses on the optimal genes associated with GBM related probes (see Supplementary Table 4).

For the GO enrichment analyses results, firstly nervous system development has been screened out. Nervous system development is a biological process related to GBM. The malignant transformation and invasive migration of glioma cells rely on basic cellular components and physical anatomical structure. Therefore, the nervous system may contain proteins that are crucial for GBM. A recent publication confirmed that MT1-MMP, a major component of nervous system development, plays an important role during the pathogenesis of GBM (Beliën et al., 1999). Nervous system development is also associated with DNA methylation. Specific patterns have been seen at the DNA methylation level in the nervous system during the development and pathogenesis of GBM. Some patterns are even shared by two groups (Numata et al., 2012). Therefore, nervous system development, as an effective biological process, can be predicted to contribute to the description of GBM, validating the efficacy and accuracy of our prediction.

Apart from that, the next enriched term calcium ion binding has also been shown to be related to GBM. Various important cells in the central nervous system and the pathogenesis of GBM-like astrocytes participate in complicated metabolite transportation from the blood to the brain. Under pathogenic conditions, glioma cells seize control of the regulation of vascular tone through the Ca^+^-dependent release of K^+^, suggesting that calcium ion binding and blood stream in the brain in pathogenic status have important clinical implications (Watkins et al., 2014). Calcium ion binding is also related to methylation. An increase in the ionic strength and a decrease in the methylation reduce the amount of calcium required for the gelation of pectin–calcium systems (Garnier et al., 1993).

### Biological Pathways Relevant to GBM Based on Optimal Genes

Apart from GO enrichment analyses, we also performed KEGG pathway analyses on such optimal genes (see Supplementary Table 4). The results of this study indicated that alcoholism is related to glioblastoma. Repurposing disulfiram (DSF) is a drug that has been widely used over the past several years to control alcoholism. DSF can inhibit the growth of GBM cells with TMZ resistance without affecting normal cells in the human central nervous system. DSF can suppress the growth and self-renewal of primary cells from GBM tumors, suggesting that an association exists between alcoholism and GBM (Triscott et al., 2012). Alcoholism is also related to the methylation alteration of transporter genes. Methylation status is further affected by alcoholism. The methylation of DAT in peripheral blood has also been validated to be a biomarker for alcohol-dependent patients (Wiers et al., 2015).

## Conclusion

We found several methylation features (sites) associated with the classification of GBM using our newly presented computational method for classifying glioblastoma subtypes on the basis of gene methylation level. Through the further functional enrichment analysis of dysmethylated genes, such as *CXCR4*, *TBX18*, *SP5*, and *TMEM22*, several potential pathogenic functions are found to participate in the initiation and progression of glioblastoma. These functions include nervous system development, intrinsic plasma membrane component, systemic lupus erythematosus, and alcoholism.

## Data Availability Statement

Two sets of methylation profiles of patients with GBM were downloaded from Gene Expression Omnibus (GEO) with the accession numbers GSE90496 and GSE109379.

## Author Contributions

TH and Y-DC designed the study. Y-HZ, ZL, TZ, and XP performed the experiments. Y-HZ, ZL, LC, DL, and HL analyzed the results. Y-HZ and ZL wrote the manuscript. All authors contributed to the research and reviewed the manuscript.

## Conflict of Interest

The authors declare that the research was conducted in the absence of any commercial or financial relationships that could be construed as a potential conflict of interest.

## Footnotes

1 http://finzi.psych.upenn.edu/R/library/stats/html/Hypergeometric.html

2 https://www.ncbi.nlm.nih.gov/geo/query/acc.cgi?acc=GPL13534

## Supplementary Material

Supplementary Table 1 Features ranked by MCFS.

Supplementary Table 2 10-fold cross-validation performance of IFS with SVM, RF, and RIPPER when using different number of features ranked by MCFS.

Supplementary Table 3 Genes corresponding to the top-ranked features by MCFS.

Supplementary Table 4 Enrichment analysis results on 1813 coding genes.
